# The effect of premedication with ketamine, alone or with diazepam, on anaesthesia with sevoflurane in parrots (*Amazona aestiva*)

**DOI:** 10.1186/1746-6148-9-142

**Published:** 2013-07-17

**Authors:** Valéria Veras Paula, Denise Aya Otsuki, José Otávio Costa Auler, Talyta Lins Nunes, Aline Magalhães Ambrósio, Denise Tabacchi Fantoni

**Affiliations:** 1 Department of Animal Science, Universidade Federal Rural do Semi-Árido, Av. Francisco Mota, 572, Bairro Costa e Silva, CEP: 59.625-900, Mossoró, RN, Brazil; 2 Laboratory of Medical Investigation/Anesthesiology (LIM/08), Faculdade de Medicina da Universidade de São Paulo, Av. Dr. Arnaldo, 455, Cerqueira César, CEP 01246903, São Paulo, SP, Brazil; 3 Department of Surgery, Faculdade de Medicina Veterinária e Zootecnia da Universidade de São Paulo, Av. Prof. Dr. Orlando Marques de Paiva, 87 CEP 05508 270, Cidade Universitária, São Paulo, SP, Brazil

**Keywords:** Sevoflurane, Ketamine, Diazepam, Parrots

## Abstract

**Background:**

Premedication is rarely used in avian species. The aim of this study was to evaluate the effect of premedication on the quality of sevoflurane induction and anaesthesia in parrots. We hypothesised that premedication would facilitate handling and decrease the minimum anaesthetic dose (MAD). Thirty-six adult parrots were randomly distributed in three groups: group S (n = 12) was premedicated with NaCl 0.9%; group KS (n = 12) was premedicated with 10 mg.kg^-1^ ketamine; and group KDS (n = 12) was premedicated with 10 mg.kg^-1^ ketamine and 0.5 mg.kg^-1^ diazepam, delivered intramuscularly. After induction using 4.5% sevoflurane introduced through a facemask, the MAD was determined for each animal. The heart rate (HR), respiratory rate (RR), systolic arterial blood pressure (SAP), and cloacal temperature (CT) were recorded before premedication (T0), 15 minutes after premedication (T1), and after MAD determination (T2). Arterial blood gas analyses were performed at T0 and T2. The quality of anaesthesia was evaluated using subjective scales based on animal behaviour and handling during induction, maintenance, and recovery. Statistical analyses were performed using analysis of variance or Kruskal-Wallis tests followed by Tukey’s or Dunn’s tests.

**Results:**

The minimal anaesthetic doses obtained were 2.4 ± 0.37%, 1.7 ± 0.39%, and 1.3 ± 0.32% for groups S, KS, and KDS, respectively. There were no differences in HR, RR, or CT among groups, but SAP was significantly lower in group S. Sedation was observed in both the premedicated S-KS and S-KDS groups. There were no differences in the quality of intubation and recovery from anaesthesia among the three groups, although the induction time was significantly shorter in the pre-medicated groups, and the KS group showed less muscle relaxation.

**Conclusions:**

Ketamine alone or the ketamine/diazepam combination decreased the MAD of sevoflurane in parrots (*Amazona aestiva*). Ketamine alone or in combination with diazepam promoted a good quality of sedation, which improved handling and reduced the stress of the birds. All protocols provided safe anaesthesia in this avian species.

## Background

For the last several years, interest in wildlife conservation has promoted an increase in the number of studies concerning the use of anaesthetics in wild birds and domestic fowl [1]. Anaesthesia in birds is challenging, and the literature describes the use of different drugs and dosages for several different species, ranging from canaries to ratites [2,3].

Research based on the study of wild birds is becoming more and more difficult. The Brazilian Institute of the Environment and Renewable Natural Resources (IBAMA) maintains rigid control over activities carried out by researchers, with the aim of preserving the lives of these animals. In contrast, many species are in danger of extinction, which makes studies of these birds essential.

The principal problems with the use of anaesthetics in birds are related to physiological, anatomical, and metabolic differences in birds compared with mammals, the lack of studies in this field, and the large spectrum of species that remain to be found.

As in other species, general anaesthetics in birds may be carried out by the use of injectable agents and inhalation [2].

Pre-anaesthetic medication is rarely indicated for small birds, as these animals can be restrained manually [4]. Nevertheless, small birds are very delicate and extremely sensitive to manual restraint, which makes pre-anaesthetic medication a viable option. Additionally, manual restraint can be extremely stressful to birds, resulting in organic reactions that may interfere with the internal stability of the organism and produce serious consequences [5].

Injectable anaesthetics are commonly used in ambulatory procedures, such as the treatment of wounds and x-ray examinations, and they may also be used as pre-anaesthetic medication or for the induction of anaesthesia [4]. Ketamine is the most frequently used agent [6] and has been used in many different species of birds [7]. The combination of ketamine and benzodiazepines is also frequently used in bird species [8].

The majority of veterinary anaesthetists prefer inhalation anaesthesia, particularly isoflurane and sevoflurane, for avian species, although this method has a disadvantage in that it requires anaesthetic delivery equipment [2]. Among the different inhalant anaesthetics, sevoflurane has several advantages, such as a lower blood-gas partition coefficient and a non-pungent odour, and it also provides rapid induction and recovery [9,10]. Both sevoflurane and isoflurane have been proven safe for use in parrots [11,12].

Within this context, the aim of this study was to evaluate the effectiveness of premedication in parrots (*Amazona aestiva*) using ketamine, either alone or in combination with diazepam, followed by the induction and maintenance of anaesthesia with sevoflurane. We hypothesised that premedication would facilitate handling and decrease sevoflurane requirements during anaesthesia.

## Methods

### Birds

Approval for the study was obtained from the local ethics committee and IBAMA (Brazilian Institute of the Environment and Renewable Natural Resources). The study utilised thirty-six Amazon parrots (*Amazona aestiva*) weighing 284 to 460 g (360 ± 37 g) that were housed at the Tietê Ecological Park (São Paulo, Brazil). Physical examinations were performed, and only birds with a good nutrition status and absence of respiratory disease were used. All procedures were performed within the park’s facilities.

### Experimental procedure

The birds were randomly distributed into three groups: the sevoflurane group (S) (n = 12) was premedicated with NaCl 0.9% as a placebo, the ketamine/sevoflurane group (KS) (n = 12) was premedicated with ketamine 10 mg.kg^-1^, and the ketamine/diazepam/sevoflurane group (KDS) (n = 12) was premedicated with ketamine 10 mg.kg^-1^ and diazepam (solubilised in propylene glycol) 0.5 mg.kg^-1^ by intramuscular injection into the pectoral musculature. All drugs were diluted in NaCl 0.9%, and the same volume (0.2 ml) was administered for every group.

### Determination of the minimum anaesthetic dose (MAD)

Fifteen minutes after premedication, anaesthetic induction was accomplished via the delivery of 4.5% sevoflurane in 100% oxygen with a flow rate of 1.5 L/min through a facemask connected to a non-rebreathing circuit. The trachea was intubated with a non-cuffed endotracheal tube (2.5 mm internal diameter). After intubation, the end-tidal sevoflurane level was reduced to 3.5% and kept constant for 5 minutes. The partial pressure of expired CO_2_ (P_E_CO_2_) and the inspired and end-tidal sevoflurane concentration were monitored throughout the experiment with a multigas monitor (Poet IQ, Critcare, Waukesha, USA) connected to the breathing circuit through an 18-gauge needle inserted into the lumen of the endotracheal tube adapter. The sampling rate was measured at 150 ml/min.

After the equilibration period, the bird was stimulated with a haemostat. The full length of the haemostat jaw was clamped to full ratchet lock to a claw for 1 minute or until gross purposeful movement was observed (such as lifting of the head and neck, flapping of the wing or kicking of the legs). When no movement was observed, the end-tidal sevoflurane concentration was reduced by 20%. The stimulus was repeated after at least 5 minutes of equilibration. Additional decrements of the end-tidal sevoflurane concentration were made (20% each time) until a purposeful movement occurred. After detecting movement, the end-tidal sevoflurane concentration was increased by 10%; after 5 minutes of equilibration, the stimulus was repeated. The mean sevoflurane concentration between that allowing movement and that preventing movement was considered the minimum anaesthetic dose [13].

### Physiologic variable and arterial blood gas monitoring

Heart rate (HR) was monitored using a stethoscope placed on the lower left lateral thoracic wall for 1 minute. Respiratory rate (RR), which was monitored by multigas monitor, was determined based on thoracic excursion for 1 minute after the induction of anaesthesia. Cloacal temperature (CT) was measured with a digital thermometer (digital clinical thermometer, Omrom, China). Systolic arterial blood pressure (SAP) was measured with a Doppler ultrasonic instrument (Ultrasonic Doppler 811 - Parks Medical Electronics, Oregon, USA) with the sensor placed in the region of the tibial artery and a paediatric cuff (2.2 cm width, approximately 40% of the bird thigh circumference) placed around the thigh.

Arterial blood (0.3 ml) samples were collected anaerobically from the superficial ulnar artery into heparinised (sodium heparin) 1 ml plastic syringes. The samples were analysed immediately using a portable analyser (i-STAT, Abbott, Illinois, USA) with cartridges measuring hydrogen potential (pH), arterial carbon dioxide partial pressure (PaCO_2_), arterial oxygen partial pressure (PaO_2_), arterial oxygen saturation (SaO_2_), base excess (BE), bicarbonate ion $\left({\mathrm{HCO}}_{3}^{-}\right)$, haematocrit (Ht), haemoglobin (Hb), sodium (Na^+^), potassium (K^+^), and ionised calcium (iCa) concentrations (EG7+, Abbott, Illinois, USA). Blood gas values were corrected based on the cloacal temperature. The i-STAT analyser was tested daily with quality tests with an external electronic simulator, as recommended by the manufacturer, prior to its use in experiments.

Each bird was positioned in a dorsal recumbent position, and its body temperature was maintained at approximately 40°C with a circulating warm water blanket (T/Pump, Gaymar, Orchard Park, NY, USA). Room temperature was maintained at 24-28°C.

During the recovery from anaesthesia, the birds were wrapped in paper so that they could rise only when their muscular coordination was sufficient to allow them to maintain a bipedal position, thus avoiding spastic movements that could cause accidental injury.

All parameters described were evaluated every 5 minutes. For statistical analysis, these parameters were considered at the following time points: baseline, before premedication (T0); 15 minutes after premedication (T1); and immediately after MAD determination (T2).

### Anaesthesia evaluation

Induction time was defined as the time from the placement of the facemask to the time at which the animal succumbed in lateral recumbency, i.e., with relaxed wings and an absence of movement.

The extubation time was defined as the time from the termination of sevoflurane administration until tracheal extubation, which occurred immediately following the recovery of increased jaw tone.

The time required to return to a bipedal position was determined as the interval from the termination of sevoflurane administration until the bird returned to an upright position.

The time to complete recovery was defined as the time between the termination of sevoflurane administration until the moment the bird returned to a bipedal position, walked in the cage, and was able to remain stable on a perch.

The degree of sedation, induction, muscle relaxation, and recovery from anaesthesia were evaluated subjectively. A score from 0 to 2 was given for each category, with a higher number indicating an improved quality of the variable (Table 1).

**Table 1:** Evaluation of the quality of sedation, induction, muscle relaxation, and recovery from anaesthesia in parrots anaesthetised with sevoflurane

**Criteria**	**Score**	**Observation**
Sedation	2	Animal is undisturbed after sedation, allows manipulation without stress and accepts the mask for the induction of anaesthesia.
	1	Animal resistant to manipulation, appears stressed and avoids the mask.
	0	Animal does not accept manipulation or mask.
Induction	2	Tracheal intubation is performed easily; animal is relaxed, and does not exhibit reflexes in response to intubation stimulus; absence of excitation.
	1	Difficulty in proceeding with tracheal intubation; orotracheal reflex and excitation observed; intubation permitted.
	0	Not possible to proceed with tracheal intubation; wing and leg movements and vocalisation observed.
Muscle relaxation	2	Complete muscle relaxation.
	1	Light muscle tonus.
	0	Intensive muscle tonus of wings and contracted pelvic members.
Recovery	2	Calm animal, without excitation or vocalisation.
	1	Smooth awakening, but animal tries to remain standing up, beating the wings.
	0	Disturbed awakening, with violent beating of wings violently and vocalization.

### Statistical analysis

The primary outcome was a decreased MAD in the premedicated groups. The sample number (12 per group) was calculated based on the published chicken sevoflurane MAD 2.2 ± 0.32% [10], a reduction of 20% in premedicated groups, and a power of 80%.

The data were tested for normality using the Kolmogorov-Smirnov test. Parametric data (cardiorespiratory and blood gas) were compared within groups and between groups by a two-way repeated measures analysis of variance (ANOVA) with the Tukey-Kramer post hoc multiple comparison test. The MAD, time for induction, and recovery were compared between groups by one-way ANOVA and the Tukey-Kramer post-hoc multiple comparison test. Non-normally distributed data (sedation, induction, muscle relaxation, and recovery scores) were compared between groups using the Kruskal-Wallis test. When appropriate, a post-hoc multiple comparison analysis was performed using Dunn’s test (SigmaStat 3.11, Systat Software Inc., San Jose, CA, USA). A P < 0.05 was considered statistically significant. The data are reported as the mean ± standard deviation unless otherwise indicated.

## Results

The MAD in the group S (2.4 ± 0.37%) was significantly higher than that in the KS (1.7 ± 0.39%, P < 0.001) and KDS (1.3 ± 0.32%, P < 0.001) groups. The values for the group KDS were significantly lower than those for the group KS (P = 0.001).

**Figure 1:**
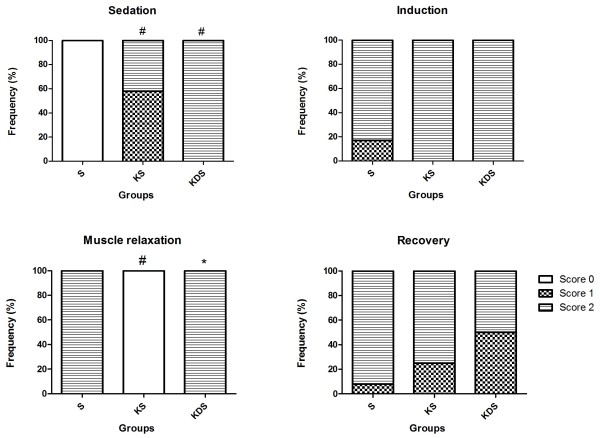
**Anaesthesia quality scores.** # P < 0.05 compared with the S group; * P < 0.05 compared with the KS group.

Sedation was observed in both premedicated groups KS and KDS. There was no difference in the quality of sedation produced by ketamine alone or associated with diazepam. The birds in the group KS showed less muscle relaxation compared with the birds in the S and KDS groups. The scores reflecting the degree of sedation, induction, muscle relaxation, and recovery from anaesthesia are shown in Figure 1.

The anaesthetic induction time was significantly shorter in the two premedicated groups (Table 2). The extubation times varied among the groups and were significantly shorter in the group KS (premedicated with ketamine alone). The time required to return to an upright position was significantly longer in the premedicated groups. The total anaesthesia times (from T0 to T2) were 52.6 ± 14.6, 67.2 ± 15.0, and 78.9 ± 11.2 for groups S, KS, and KDS, respectively. This time was significantly shorter in the group S than in the KS and KDS groups (P = 0.036 and P < 0.001, respectively).

**Table 2:** Times for variables associated with anaesthesia (mean ± SD)

**Group**	**Time (s)**			
	**Induction**	**Extubation**	**Upright**	**Recovery**
**S**	82 ± 29.7	56 ± 23.8	129 ± 63.8	348 ± 92.4
**KS**	46 ± 13.7^#^	48 ± 21.1	239 ± 120.6^#^	575 ± 192.3
**KDS**	38 ± 12.7^#^	97 ± 38.5^#*^	258 ± 97.5^#^	909 ± 331.5^#*^

# P < 0.05 compared with S; * P < 0.05 compared with KS.

The RR decreased significantly at T1 in the KS and KDS groups and at T2 in all groups (Table 3). SAP in the group S was significantly lower at T2 than at T0 and T1. At T2, the SAP in the group S was also significantly lower than that in the KS and KDS groups. The CT in the group S decreased at T2, while in the KS and KDS groups, the CT was significantly lower at T1 and T2.

**Table 3:** Physiological parameters (mean±SD)

**Parameter**	**Group**	**T0**	**T1**	**T2**
HR (beat.min^-1^)	S	183 ± 33	164 ± 23	195 ± 34^†^
	KS	180 ± 38	167 ± 33	193 ± 54
	KDS	174 ± 33	163 ± 24	180 ± 32
RR (breaths.min^-1^)	S	93 ± 26	79 ± 31	31 ± 06^Δ†^
	KS	65 ± 31^#^	29 ± 08^#Δ^	22 ± 06^Δ^
	KDS	71 ± 28^#^	31 ± 09^#Δ^	21 ± 07^Δ^
SAP (mmHg)	S	170 ± 29	176 ± 26	136 ± 21^Δ†^
	KS	175 ± 19	177 ± 22	163 ± 31^#^
	KDS	175 ± 24	175 ± 37	168 ± 25^#^
CT (°C)	S	42 ± 0.52	41.5 ± 0.86	40 ± 1.28^Δ †^
	KS	41.8 ± 0.69	41 ± 0.5^Δ^	40.4 ± 0.94^Δ^
	KDS	41.7 ± 0.71	40.2 ± 0.57^Δ #^	40.3 ± 0.84^Δ^

*HR* heart rate, *RR* respiratory rate, *SAP* systolic blood arterial pressure, *CT* cloacal temperature; T0: before premedication; T1: 15 min after premedication; T2: after MAD; # P < 0.05 compared with S; Δ P < 0.05 compared with T0; † P < 0.05 compared with T1.

The pH decreased significantly at T2 compared with T0 in the group KDS, while the values of PaCO_2_, PaO_2_, SaO_2_, BE, and ${\mathrm{HCO}}_{3}^{-}$ increased significantly at T2 in the three groups compared with T0 (Table 4).

**Table 4:** Arterial blood gases and electrolytes (mean±SD)

	**Group**			
	**Moment**	**S**	**KS**	**KDS**
pH	T0	7.436 ± 0.042	7.458 ± 0.052	7.463 ± 0.056
	T2	7.410 ± 0.053^Δ^	7.433 ± 0.057^Δ^	7.386 ± 0.055^Δ^
PaCO_2_ (mmHg)	T0	22.1 ± 3.45	22.0 ± 4.56	22.1 ± 3.63
	T2	39.7 ± 5.33^Δ^	34.1 ± 6.4^Δ^	40.1 ± 7.77^Δ^
PaO_2_ (mmHg)	T0	100.2 ± 6.94	98.3 ± 8.14	96.0 ± 7.15
	T2	391.8 ± 63.74^Δ^	324.4 ± 112.3^Δ^	372.0 ± 85.48^Δ^
SaO_2_ (%)	T0	96.2 ± 1.03	96.3 ± 1.07	96.2 ± 1.19
	T2	100.0 ± 0.0^Δ^	99.9 ± 0.29^Δ^	100.0 ± 0.0^Δ^
BE (mmol.L^-1^)	T0	- 8.7 ± 2.31	−7.6 ± 3.42	−7.4 ± 3.68
	T2	0.3 ± 4.41^Δ^	−1.3 ± 3.52^Δ^	−0.8 ± 2.69^Δ^
$\begin{array}{l} {\mathrm{HCO}}_{3}^{-}\\ \left(\text{mmol}.L^{-1}\right) \end{array}$	T0	14.3 ± 1.95	15.1 ± 3.2	15.1 ± 3.03
	T2	24.7 ± 3.78^Δ^	22.2 ± 3.30^Δ^	23.3 ± 2.80^Δ^
Ht (%)	T0	37.6 ± 6.33	38.8 ± 8.49	36.8 ± 5.34
	T2	37.4 ± 4.12	37.6 ± 7.42	37.8 ± 3.66
Hb (g.dL^-1^)	T0	12.9 ± 2.17	13.2 ± 2.88	12.5 ± 1.78
	T2	12.8 ± 1.50	12.8 ± 2.51	12.9 ± 1.29
Na^+^ (mmol.L^-1^)	T0	147.0 ± 1.35	147.2 ± 2.86	147.6 ± 2.15
	T2	145.6 ± 1.88	146.3 ± 3.55	147.7 ± 2.10
K^+^ (mmol.L^-1^)	T0	3.5 ± 0.36	3.9 ± 1.15	3.6 ± 0.56
	T2	3.8 ± 0.53	4.5 ± 1.57	3.9 ± 0.37
iCa (mmol.L^-1^)	T0	0.7 ± 0.29	0.8 ± 0.31	0.7 ± 0.23
	T2	0.8 ± 0.19	0.8 ± 0.29	0.8 ± 0.24

*PaCO*_*2*_ arterial carbon dioxide partial pressure, *PaO*_*2*_ arterial oxygen partial pressure, *SaO*_*2*_ arterial oxygen saturation, *BE* base excess, *HCO*_*3*_ bicarbonate ion concentration, *Ht* haematocrit, *Hb* haemoglobin concentration, *Na*^*+*^ serum sodium concentration, *K*^*+*^ serum potassium concentration, *iCa* serum ionised calcium concentration, *T0* before premedication, *T2* after MAD; Δ P < 0.05 compared with T0.

After the determination of the MAD, we observed a P_E_CO_2_ of 43.4 ± 4.77 mmHg in the group S, 40.4 ± 3.78 mmHg in the group KS, and 45.2 ± 0.84 mmHg in the group KDS. The PaCO_2_, analysed at the same time, was 39.7 ± 5.33 mmHg in the group S, 34.1 ± 6.04 mmHg in the group KS, and 40.1 ± 7.77 mmHg in the group KDS.

## Discussion

The use of ketamine, ketamine-diazepam, and sevoflurane proved to be adequate for clinical restraint and anaesthesia in parrots. In the present study, premedication of parrots with ketamine alone or in combination with diazepam promoted sedation, decreased struggling during induction, and also reduced the MAD.

In mammals, minimum alveolar concentration (MAC) has been used as the index of potency for inhalant anaesthetics [14]. In birds, although this term is inappropriate for this species because they do not possess alveolus lung cavities, the potency of these inhalants has been determined by methods similar to those used in mammals. Parameters such as Minimum Anaesthetic Concentration [10], Effective Dose (ED_50_) [15], and Minimum Anaesthetic Dose [13] have been used to express the potency of inhalant anaesthetics in birds.

In the present study, the MAD obtained with the use of sevoflurane was 2.3 ± 0.31%, which is lower than the value observed in a previous study of parrots [16]. However, it is similar to the value of 2.35% obtained in thick-billed parrots [12] and the values obtained in other species, such as 2.21% in chickens [10], 2.36 ± 0.46% in dogs [17], 2.8% in cats [18], and 2.3% in horses [19].

Several factors may alter the MAC of inhalant anaesthetics in mammals, including animal age, temperature extremes, circadian rhythms, hypoxemia, hypotension, and the presence of other chemical elements such as sedatives, narcotics, and tranquilisers [14]. In this study, the MAD of sevoflurane diminished significantly when the parrots were pre-medicated either with ketamine or a combination of ketamine/diazepam (decreased by 25% and 45.8%, respectively).

Although there were no differences between the groups KS and KDS, the quality of sedation was higher in the premedicated groups. Indeed, the parrots that had not been premedicated were difficult to handle because they refused to accept manual restraint and fought against the placing of the induction mask, thus making the performance of the medical procedure dangerous for both the anaesthetist and the animal. In contrast, the birds that were premedicated were easy to handle, and the placement of the induction mask was permitted without any indication of stress. Thus, one of the main findings of this study is that premedication improved the quality of sedation and reduced the stress to the birds.

The quality of intubation did not vary among the groups, and no difference in the ease of introducing the endotracheal tube was noted. In all birds, the induction was considered to be free of any type of excitation after the mask had been placed. The use of ketamine and ketamine/diazepam decreased the induction time (44% and 54% in KS and KDS groups, respectively), presumably because these agents potentiate inhalant anaesthetics [20]. The induction time observed in the birds that did not receive premedication was shorter than those reported for thick-billed parrots (3 min with 5% sevoflurane in oxygen 1 L/min) [12] and red-tailed hawks (3.6 minutes with 5.75% sevoflurane in oxygen 1 L/min) [21]. However, comparing the times necessary for induction across these studies is difficult due to differences in species and the definition of time to induction. The oxygen flow and the anaesthetic inspiratory concentration may also influence the induction time [9].

In our analysis, the extubation and recovery times were both significantly different among the groups. The use of benzodiazepines can increase recovery time because these molecules increase muscle relaxation, have sedative and hypnotic properties, and also potentiate dissociative anaesthetics. Diazepam is frequently used because it produces similar muscle relaxation but has a longer duration of sedation than midazolam [22,23]. Midazolam may be a better choice due to its water solubility, but previous experience has shown no clinical muscle damage due to the injection of a small volume of propylene glycol diazepam.

In the present study, the quality of recovery was similar in the three groups analysed; however, we observed that the recovery process was more gradual in the premedicated groups. Very rapid recovery has been observed in birds that were anaesthetised with inhalants only [24]. Although the time required to return to a bipedal position differed significantly among groups, from a clinical point of view, they were very similar, ranging from 2.15 min in the group S to 4.3 min in the group KDS. Wrapping the birds in paper contributed to the uneventful recovery. The time necessary for complete recovery in the group sevoflurane was similar to those reported in a different study in the same species (4.47 min) [16] and in roosters (5.5 min) [25]. In the premedicated birds, the recovery times were 9.6 ± 3.2 minutes (ketamine group) and 15.2 ± 8.9 minutes (ketamine/diazepam group). Premedication with ketamine alone or in association with diazepam increased the length of MAD determination, requiring more end-tidal sevoflurane concentration decrements and equilibrium times, thus resulting in longer times from T0 to T2. This longer anaesthesia time is unlikely to have affected the length of recovery from sevoflurane, which has a low blood/gas partition coefficient (0.69) [26], but the possibility of such interference cannot be ruled out.

When comparing time T0 to T1, we observe that the use of premedication significantly decreased the respiratory rate, which may be explained by the tranquilising and sedative effects of the agent used. In dogs, ketamine causes a transient lowering of the respiratory rate, minute volume, and PaO_2_, with an initial increase in PaCO_2_[27]. No depression of the respiratory rate was observed in domestic chickens injected with a combination of 20 mg.kg^-1^ of ketamine and 2 mg.kg^-1^ of diazepam [28]. Ketamine causes fewer cardiovascular and respiratory effects than other anaesthetics [29]. In our study, the basal respiratory rates of the birds in all three groups studied were elevated, most likely reflecting the hyperventilation caused by stress during restraint. This could be considered a limitation of the study itself, given that the study involves non-domesticated animals. Because these birds are easily stressed and difficult to handle, the use of pre-anaesthetic medication is even more justifiable. A decrease in respiratory rate was observed in all of the groups analysed after determining the MAD (T2) in comparison with baseline (T0), and there were no significant differences among the groups, suggesting that premedication does not significantly potentiate this decrease. The respiratory rates measured after the application of the anaesthetic (T2) were similar to those observed in another study in the same species [16] and in thick-billed parrots [12] and higher than the values observed in chickens [30].

The HR and SBP were similar in all three groups at T1. However, in group S, the heart rate increased significantly during sevoflurane administration. This increase is likely a result of a significant decrease in blood pressure related to the higher concentration of inhaled anaesthetic verified in this group at this time point. Similar results were observed in chickens anaesthetised with sevoflurane, in which the HR increased and the SBP decreased with increasing inhaled concentrations [30].

Although a circulating-water warming blanket was utilised, it was not sufficient to prevent a temperature decrease in all groups at T2. The circulating-water blanket minimises heat loss but not as efficiently as the forced-air warmer system [31]. In the KS and KDS groups, the decrease in CT was observed immediately following premedication, but at T2, the CT was similar in all groups, showing that premedication did not enhance heat loss over time. The longer anaesthesia time observed in the KDS group also did not increase heat loss. Temperature may alter anaesthetic requirements [14], but it is unlikely that the differences observed at T1 contributed to the decreased MAD in the KDS and KS groups.

The analyses of the blood gas and electrolytes in non-anaesthetised parrots are similar to the data obtained under similar conditions in other studies involving avian species [8,32-35]. Even with the appropriate physical restraint of the birds, the stress of this procedure causes hyperventilation, which produces a decrease in PaCO_2_.

Inhalant anaesthetics cause respiratory depression, depending on the dose, as reported by a large number of authors and evidenced by an increase in PaCO_2 _[34]. Hypercapnia has been shown to induce arrhythmia in ducks anaesthetised with halothane [36]. These effects may be due to the myocardium-sensitising effect of halothane and the sympathomimetic effect of CO_2_. Sevoflurane is likely less apt to induce arrhythmias because it does not sensitise the myocardium to catecholamines [37]. In our study, the PaCO_2_ at the moment in which the MAD was determined was significantly increased compared with the baseline value in all groups but was compatible with the results obtained in chickens (46 mmHg with 2.2% sevoflurane) [30] and thick-billed parrots (38.6 mmHg with 2.35% sevoflurane) [12]. Moreover, the PaCO_2_ values in this study were lower than those reported in ducks (57.2 ± 4.2 and 93.9 ± 11.8 mmHg using 1.0 and 1.5 MAD isoflurane, respectively) [34] and parrots (55.87 mmHg with 3.44% sevoflurane) [16].

It must be noted that the significant increase in PaCO_2_ observed following the administration of sevoflurane was due, in part, to the fact that the parrots suffered from respiratory alkalosis at T0 as a result of the hyperventilation caused by restraint-related stress. Thus, the difference in the concentrations at T0 and T2 may seem to be much greater than it really is. In contrast, it is virtually impossible to collect blood samples from these birds in a stress-free situation because they are not domesticated, which undoubtedly limits our ability to accurately evaluate baseline PaCO_2_ values.

In this study, the P_E_CO_2_ values exceed the PaCO_2_ values by approximately 5 mmHg, similar to the results observed in grey parrots anaesthetised with isoflurane [38]. In mammals, the exact opposite occurs; the P_E_CO_2_ is lower than the PaCO_2 _[39,40]. This result is explained by the fact that in mammals, the airflow in the alveolus is bidirectional and the physiological dead space has an important effect on the P_E_CO_2_, whereas in birds, parabronchial air flow is unidirectional throughout the respiratory cycle [3]. The cross-current gas exchange in parabronchi also contributes to a higher gas exchange efficiency than in mammals [41]. Considering the anatomical and physiological differences between birds and mammals, capnography is a reliable and important tool for monitoring the PaCO_2_ and the degree of respiratory depression during anaesthesia in parrots.

The small size of the birds (360 ± 37 g) can lead to the assumption of inaccuracy in capnographic measurements. However, the sampling rate (150 ml/min) did not exceed the minute ventilation calculated at approximately 160–230 ml (RR × (22.9 × weight^1.08^)) [42]. The fresh gas flow was also 10 times the sampling rate. Moreover, the capnogram inspiratory CO_2_ was always near zero, indicating no reinhalation in the system. Upon the increase in sevoflurane delivery, the obtained inspiratory sevoflurane was higher than the expiratory concentration, reaching values close to equilibrium after the stabilisation period. Still, the possibility of some degree of inaccuracy in the P_E_CO_2_ measurements should be recognised as a limitation of this study.

## Conclusions

Ketamine, delivered alone or in combination with diazepam, considerably decreased the MAD of sevoflurane in parrots (*Amazona aestiva*). Ketamine alone or in combination with diazepam promoted a good quality of sedation, thereby improving handling and reducing the stress of the birds. All protocols provided safe anaesthesia in this avian species.

## Abbreviations

BE: Base excess in mmol.L^-1^; CT: Cloacal temperature in °C; group KDS: group ketamine/diazepam/sevoflurane; group KS: group ketamine/sevoflurane; group S: group sevoflurane; Hb: concentration of haemoglobin in g.dL^-1^; ${{\mathrm{HCO}}_{3}}^{-}$: concentration of bicarbonate ions in mmol.L^-1^; HR: Heart rate; Ht: Haematocrit in %; IBAMA: Brazilian Institute for the Preservation of the Environment and Renewable Natural Resources; iCa: Serum concentration of calcium in mmol.L^-1^; K+: Serum concentration of potassium in mmol.L^-1^; MAC: Minimum alveolar concentration; MAD: Minimum anaesthetic dose; Na+: Serum concentration of sodium in mmol.L^-1^; PaCO_2_: Partial arterial carbon dioxide pressure in mmHg; PaO_2_: Partial arterial oxygen pressure in mmHg; RR: Respiratory rate; SaO_2_: Arterial oxygen saturation in %; SBP: Systolic blood pressure in mmHg; T0: Time before premedication; T1: 15 minutes after premedication; T2: After MAD determination.

## Competing interests

The authors declare that they have no competing interests.

## Authors’ contributions

JOCAJ and DTF designed the study, evaluated the data, and obtained the funding for the study. VVP and DAO conducted the experiments, collected all samples, performed the blood gas analysis, and helped to write the manuscript. TLN and AMA helped with the editing and revision of the manuscript. All authors read and approved the final manuscript.
